# Recent Advances in the Endoscopic Management of Gastro-esophageal Reflux Disorder: A Review of Literature

**DOI:** 10.7759/cureus.26218

**Published:** 2022-06-22

**Authors:** Kunal Ajmera, Nigil Thaimuriyil, Nihar Shah

**Affiliations:** 1 Epidemiology, George Washington University, Washington, DC, USA; 2 Internal Medicine, Baptist Hospitals of Southeast Texas, Beaumont, USA; 3 Gastroenterology, Sarasota Memorial Hospital, Sarasota, USA

**Keywords:** gastroesophageal reflux disease (gerd), proton-pump inhibitors (ppi), lnf, endoscopic treatment, stretta, tif

## Abstract

Gastro-esophageal reflux disorder (GERD) is the most common gastrointestinal tract disorder with high morbidity and heavy economic burden. Despite being treated with high-dose proton-pump inhibitors or H2 receptor blockers, a considerable percentage of patients have GERD that is only partially controlled or refractory. The majority of these patients forego surgical treatment for fear of adverse outcomes, putting them at a financial disadvantage and causing loss of productivity. Untreated GERD is the sole known risk factor for developing Barrett's esophagus and esophageal adenocarcinoma if left untreated. With the advancement in therapeutic modalities in recent years, and given the issues such as medication compliance, the risk of adverse events with long-term antisecretory treatment, and fear of undergoing surgical treatment, endoscopic treatments such as Stretta and transoral incisionless fundoplication (TIF) have become a safe, cost-effective, and resilient option for the treatment of refractory GERD. Patients with refractory GERD ineligible for endoscopic therapies due to a large hiatal hernia can have their hiatal hernia corrected simultaneously with TIF (C-TIF). For the treatment of refractory GERD, endoscopic therapy is a viable and compelling option. Endoscopic therapies for refractory GERD patients are highly recommended due to their reproducible and standardized results as well as the potential to address the fundamental mechanical issue.

## Introduction and background

According to the Montreal consensus meeting, gastro-esophageal reflux disorder (GERD) is a chronic illness caused by the retrograde movement of stomach contents into the esophagus, causing mucosal injury [1,2]. It is a multifaceted illness with troublesome symptoms and complications that cause considerable morbidity. With a prevalence rate of 18.1-27.8% in North America, it is the most commonly diagnosed digestive tract disorder, imposing a substantial financial strain on the economy (12 billion USD for the year 2004) [3]. Because drugs like proton-pump inhibitors (PPIs) and H2 receptor blockers are readily available over the counter, the actual incidence/prevalence rate may be substantially higher. According to scientific analysis, the percentage of young patients with GERD is on the rise, particularly in the 30-39 age group [4]. This review article provides a thorough and up-to-date overview of the current developments in the endoscopic management of refractory GERD. Researchers have outlined what characteristics make endoscopic procedures an ideal treatment of choice over life-long PPIs or surgical treatment for certain patients.

## Review

Clinical presentation

Clinical manifestation of GERD is classified into either esophageal or extra-esophageal symptoms. Heartburn and regurgitation are the most prominent esophageal symptoms, defined as a burning sensation in the retrosternal area and the perception of refluxed stomach content moving into the hypopharynx or oral cavity. Chest pain unrelated to the heart, water brash, belching, and dysphagia are some of the other complaints. Peptic stricture, esophageal ulceration, Barrett's esophagus (BE), and esophageal cancer (EAC) can all result from prolonged untreated esophageal symptoms [1,2]. Cough, post-nasal drip, dental erosion, pharyngitis, sore or burning throat, gingivitis, halitosis, aspiration, snoring, nightmares, and sleep disturbance are some of the atypical and extra-esophageal signs and symptoms of GERD [1,2].

In westernized countries, there are three phenotypic presentations of GERD: a) non-erosive reflux disease (NERD), which represents 60-70% of patients; b) erosive esophagitis (EE) (30%); and c) BE (6-8%) [1,5]. The presence (in EE) or absence (in NERD) of mucosal breaks at endoscopy is the key distinction between NERD and EE [1-3]. The Los Angeles classification is used to determine the severity of EE (grade A to grade D with increasing severity) [6]. Risk factors for GERD can be divided into modifiable or non-modifiable. Smoking [7], non-steroidal anti-inflammatory drug use [7], central obesity [7-10], and moderate/high coffee/tea intake [11] are modifiable risk factors, whereas non-modifiable risk factors include age between 35 and 59 [11], sex (women) [4], or genetic factors [12]. 

Management

Management of GERD often incorporates lifestyle modifications and pharmacological (PPIs, H2 receptor blockers), endoscopic, or surgical intervention. Evidence for the value of lifestyle modifications is available only for weight loss, elevating the head of the bed while resting, and avoiding consuming food at least 3 hours before sleep [13-15]. Antisecretory medications are by far the most effective and the cornerstone of medical treatment for chronic GERD; nonetheless, a substantial proportion (20-40%) encounter issues with medication adherence, partially controlled or refractory symptoms, and quality of life (QoL) [16-18]. Long-term antisecretory medicines have been linked to dementia, chronic renal disease, myocardial infarction, malabsorption, pneumonia, osteoporosis, and *Clostridium difficile* infection [19]. Chemical acid suppression cannot correct the underlying mechanical problems like hiatal hernia or increasing lower esophageal sphincter (LES) pressure, which is one of the reasons why patients report non-PPIs responding to GERD. Mechanical repair with endoscopy or surgery to restore normal anatomy and recreate an antireflux barrier is necessary to fix the mechanical issue and allow for normal gastrointestinal (GI) tract function. Other causes such as achalasia cardia, eosinophilic esophagitis, gastroparesis, reflux-like dyspepsia, and other esophageal dysmotility should be ruled out first in patients who do not respond to antisecretory medications [20]. Historically, laparoscopic or endoscopic treatments for GERD have been both appealing and controversial. The latest developments on non-medicinal procedural treatment for refractory GERD and those seeking definitive treatment for underlying mechanical difficulties are compiled here.

A) Laparoscopic Nissen Fundoplication (LNF)

LNF is a standard surgical technique and is the currently recommended treatment for patients with refractory GERD. It entails constructing a peri-esophageal ring around the gastroesophageal junction and bolstering the LES by encircling it with the mobilized gastric fundus, either entirely (Nissen 3600) or partially (in modified LNF with the anterior (Dor, 1800) or posterior (Toupet 270°) fundoplication). LNF is superior to conventional fundoplication because it produces fewer defective plications, incisional hernias, and lower mortality [21,22]. Dysphagia and inability to belch are less frequent after Dor than after LNF, while bloating and retching are less prevalent after Toupet than after LNF. For normal-weight GERD patients who qualify for surgery, Toupet is currently the procedure of choice [23]. Other adverse events after LNF include the development of diarrhea, flatulence, or discomfort related to delayed gastric emptying, reoperation ​​in up to 15% of patients, and symptom recurrence in up to 62% of patients 11-13 years after surgery [22,24]. The incidence rate of esophageal acid exposure (EAE) and esophagitis, patient satisfaction, dilations, and reoperation rate were not different between full-thickness and modified partial-thickness fundoplication [25]. According to recent studies, the majority (>95%) of refractory GERD or PPI non-responders (20-40%) are unwilling to undertake surgical treatment ("treatment gap"), probably due to concern of post-surgery adverse effects, surgery failure, need for repeat surgery, or requirement for drugs [20]. ​​

​B) Endoscopic Treatments

Endoscopic treatments have been available for over a decade and are progressively gaining popularity to fill the void for these refractory "treatment gap" GERD patients. They are cost-effective and a safer alternative to life-long PPIs or surgical treatment [26]. Cumulative data from various clinical trials and meta-analysis reports have asserted endoscopic procedures' superiority, safety, and efficacy over PPIs and surgical management. The endoscopic antireflux devices currently available include a radiofrequency ablation device (Stretta, Mederi-RF) and endoscopic fundoplication devices such as transoral incisionless fundoplication (TIF, EsophyX). Table 1 illustrates the inclusion and exclusion criteria for selecting an optimal candidate for TIF and Stretta procedures.

**Table 1:** Inclusion and exclusion criteria for TIF and Stretta procedures. *In axial length or greatest transverse dimension. **Daily troublesome regurgitation is defined as mild symptoms occurring two or more days a week, or moderate to severe symptoms occurring more than one day a week as per the Montreal consensus definition [1] and/or atypical/extra-esophageal GERD symptoms (Montreal criteria). GERD, gastro-esophageal reflux disorder; PPI, proton-pump inhibitor.

Inclusion Criteria	Exclusion Criteria
Age 18+	BMI >35 ​​ kg/m^2^, pregnancy
GERD for >1 year	Esophageal motility disorder
Hiatal hernia <2 cm*	Hiatal hernia >2 cm*
Hx daily PPIs for >6 months	Esophageal motility disorder
Bothersome GERD symptoms on daily PPIs**	Esophageal ulcer/stricture or Barrett’s esophagus (>2 cm)
Hill grade I-II valve	Hill grade valve III or IV Los Angeles grade C or D esophagitis
Proven GERD by endoscopy, ambulatory pH, or barium swallow testing	On immunosuppressive therapy
	Portal hypertension and/or varices
	Hx of gastric or esophageal surgery
	Severe gastric paralysis
	Coagulation disorder

Xie et al. [27] conducted a meta-analysis (of 10 randomized controlled trials (RCTs) conducted in the United States, the Netherlands, and Sweden) comparing efficacy between TIF, Stretta, and PPI groups and reported statistically significant improvements in GERD-HRQL (health-related quality of life) score (TIF = Stretta > PPIs), heartburn score (TIF > Stretta > PPIs), EAE (Stretta > PPIs > TIF), and esophagitis (TIF > Stretta) (up to 1 year). The endoscopic procedures that are currently available are discussed below. 

*i) Transoral Incisionless Fundoplication*****

TIF has undergone substantial adaptations since its introduction and FDA clearance in 2007. TIF 2.0, which is currently in use, was first introduced in 2009. The EsophyX device is inserted into the esophagus through the mouth in TIF2.0 and positioned at the junction of the stomach and esophagus. A small hiatal hernia (≤2 cm) is reduced if present by engaging suction (invaginator) and positioning the esophagus below the diaphragm. A full-thickness tissue fold at the gastroesophageal junction is retracted, wrapped, and anchored using SeorsaFuse implantable fasteners - equivalent to 3.0 sutures - delivered across the tissue to complete the plication. The valve is stretched with a single device insertion, and several fasteners (12-20) are delivered. The TIF operation replaces the antireflux barrier's significant components, resulting in a tight 3-5 cm valve surrounding the distal esophagus below the diaphragm. A higher number of fasteners foreshadows better results and improves durability by reducing the continual force on the fasteners caused by esophagus and stomach mucosa [28].

Meta-analysis: A meta-analysis comparing 18 studies (RCTs and prospective observational studies) involving 963 patients by Huang et al. [29] demonstrated a clinically significant improvement in GERD-related symptoms, reduction in EAE time (p-value = 0.02), and reduction in PPI dosage when resumed over long-term post-TIF. In three RCTs (n = 71), there was no reduction in reflux episodes post-TIF procedure compared to PPIs' use (p-value 0.16) and in two RCTs (n = 150) reduction in total reflux episodes was statistically significant (p-value < 0.05). Nineteen severe adverse events in 16 studies comprising 781 participants were reported in this study. They were esophageal perforations (n = 17), post-TIF bleeding (n = 5), pneumothorax (n = 4), need for intravenous antibiotics (n = 1), and severe epigastric pain (n = 1). One death from unknown etiology was also reported 20 months after the TIF procedure.

TIF was found to be superior to LNF in boosting LES pressure (p-value > 0.05) and enhancing the HRQL (p-value > 0.05) in another meta-analysis by Richter et al. [30], which included seven RCTs with 1128 participants. In comparison, LNF outperformed TIF in terms of controlling EAE (p-value > 0.05) and lowering esophagitis (p-value > 0.05). Because data on adverse events were not consistently provided, no meta-analysis for adverse events was performed. Even though TIF was found to be inferior to LNF or PPI in terms of controlling EAE and minimizing esophagitis (statistically not a significant difference), QoL was greatly enhanced following TIF. The crucial question is how much pH regulation is actually required. Would it be more important than the patient's QoL if it is critical?

**Figure 1:**
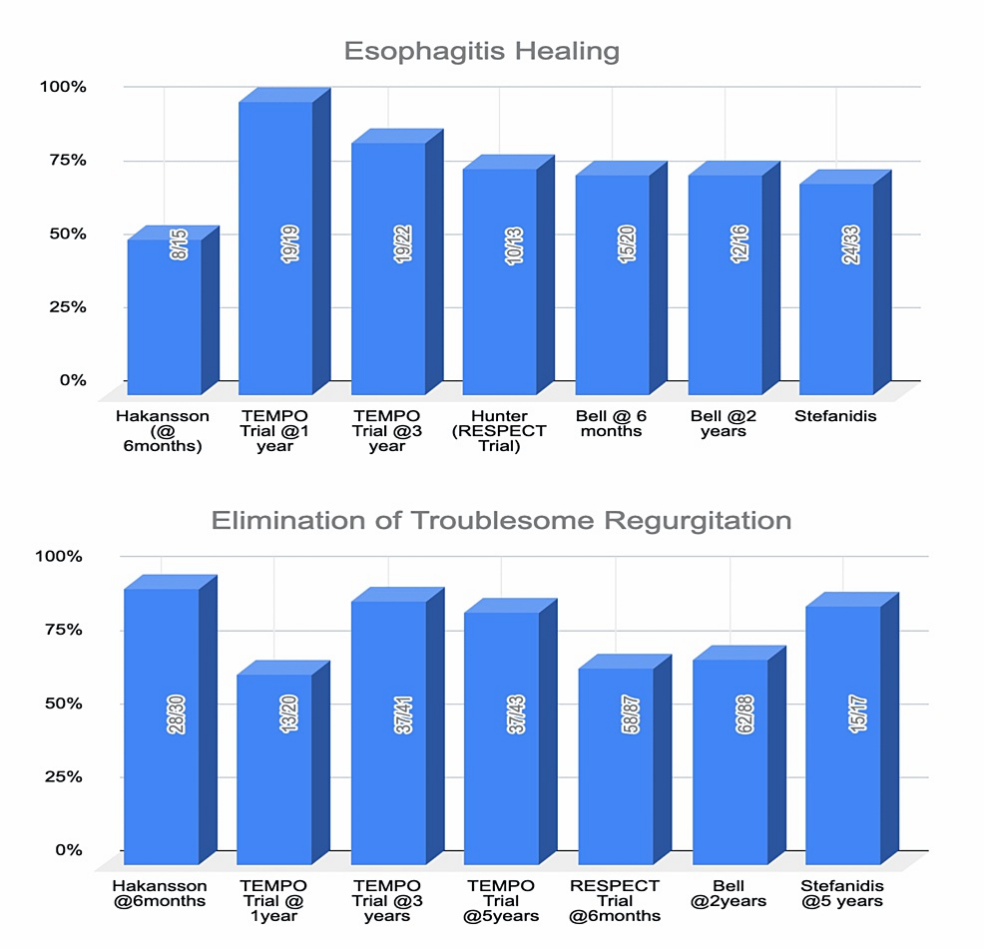
Graphical representation of comparison between esophagitis healing and elimination of troublesome regurgitation after TIF. TIF, transoral incisionless fundoplication.

**Figure 2:**
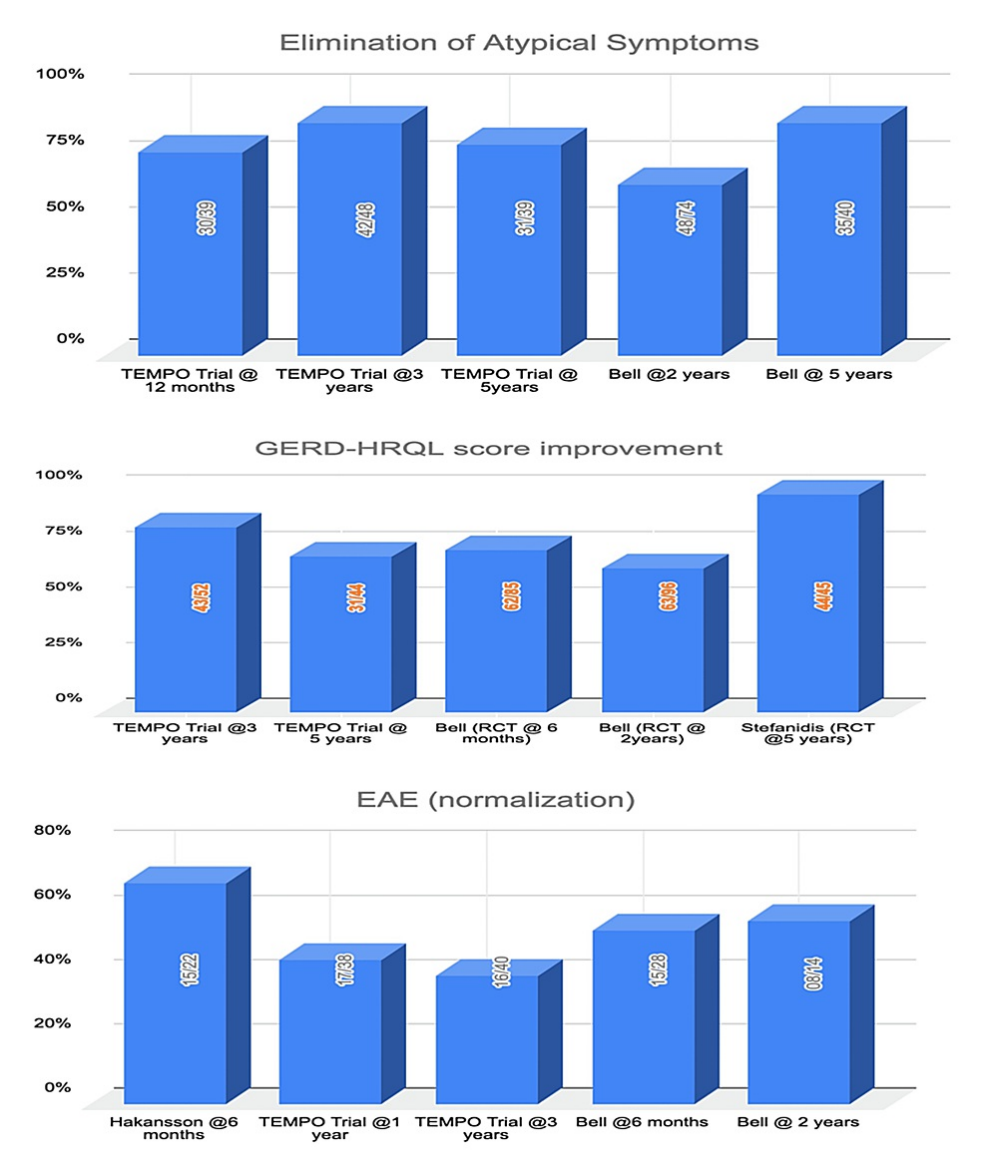
Graphical representation of comparison between elimination of atypical symptoms, GERD-HRQL score improvement, and EAE normalization post-TIF. RCT, randomized controlled trial; GERD, gastro-esophageal reflux disorder; HRQL, health-related quality of life; EAE, esophageal acid exposure; TIF, transoral incisionless fundoplication.

Clinical trials: Upon review of data from five clinical trials (with a follow-up period of six months to five years post-TIF), healing of esophagitis, EAE normalization, elimination of troublesome regurgitation, and elimination of atypical symptoms (Figures 1, 2) were found to be significantly improved in the TIF group compared to sham/PPI group [31-38]. Daily PPI usage was either completely eliminated or was resumed at a lower dose after TIF compared to baseline (Table 2).

**Table 2:** Number of participants off of PPIs at a certain follow-up period. PPI, proton-pump inhibitor.

Off PPI	Hakansson [31]	TEMPO [36-38]	Bell [33]	Stefanidis [35]
6-month follow-up	13/22		89/100	
12-month follow-up		10/60		
24-month follow-up			69/98	
3-year follow-up		14/52		
5-year follow-up		15/44		32/44

The TIF EsophyX vs. Medical PPI Open-Label Trial (TEMPO) trial was a multicenter, prospective, randomized study that was initially designed to follow up with the study participants for one and three years post-TIF and later extended to five years, which showed improvement in GERD-HRQL score in up to 70% (31/44) of the study participants at the end of five years. Elimination of troublesome regurgitation was seen in 86% (37/43) of the participants, and elimination of atypical symptoms was found in 80% (31/39) of the study participants at the end of five years. On average, 21 ± 4 fasteners were used in the procedure, likely the reason for only three out of 60 patients requiring reoperation (2 LNF, 1 Dor) at the end of the five-year follow-up period. The reoperation rate of 5% after TIF was similar to those in the published literature for LNF. No serious adverse events were reported at the end of the follow-up period. Cost analysis over a period of two years of 2734 LNF and 73 TIF patients from the Optum database showed that post-LNF average total accrued cost of care was around 99,256 USD/patient vs. 71,691 USD/patient after TIF. Cost analysis for the subgroup of the most refractory PPI group was even more impressive with an average total accrued cost of care coming down at 124,000 USD/patient after LNF vs. 66,000 USD/patient over the period of two years [36-38]. 

In a recently published clinical trial in 2018, Testoni et al. [39] followed the study participants after TIF for the longest 10-year period. Trial results were promising and showed that 86.7% of patients stopped or halved antisecretory therapy at two years of follow-up. The number increased to 91.7% at 10 years of follow-up for the same parameter. Heartburn and regurgitation symptoms also significantly improved consistently at two years, three years, and again at seven years and 10 years of follow-up. Out of 51 study participants, one patient developed intraoperative pneumothorax and two other patients developed postoperative pneumothorax. No other serious adverse events were reported [39].

*ii) Stretta*****

The Stretta technique, first introduced in 2000, entails performing an upper gastrointestinal endoscopy and transferring targeted radiofrequency (RF) energy circumferentially to the dysfunctional LES. During a series of 1-minute treatment cycles, the system uses low-power RF energy to generate low tissue temperatures in order to remodel the musculature of the LES and gastric cardia. The treatment reduces tissue compliance and transient LES relaxations, restoring the LES's natural barrier function and minimizing spontaneous regurgitation induced by transitory sphincter relaxations [40].

Many peer-reviewed studies, including comprehensive meta-analyses and RCTs, have provided data on the safety and efficacy of Stretta treatment. Improvement in GERD-HRQL score, reduction in PPI usage from baseline, improvement in basal LES pressure, reduced EAE, and the safety of the Stretta technique have been reliably established in both short- and long-term follow-ups [41-47].

Meta-analysis: A meta-analysis of 28 studies (RCTs and cohort) by Fass et al. [41] that followed 2468 participants for a mean period of little over two years showed statistically significant improvement in HRQL score, almost 50% reduction in PPI use from baseline, reduction in esophagitis, reduced EAE, and improvement in LES pressure. At an adverse event rate of only 0.93% (vs. 7.18% for the LNF), small erosions and mucosal lacerations were the most common adverse events followed by subcutaneous emphysema. No incidence of esophageal stricture post-Stretta procedure was reported. All the results were consistent and in line with previously published results [41]. Similar results from a meta-analysis of 18 studies following 1441 patients were documented by Perry et al. [42].

Clinical trials: Various clinical trials have demonstrated similar results on the efficacy and safety of the Stretta procedure. Corley et al. (2003) [43] reported the first prospective, randomized, double-blinded, multicenter study of 64 patients with GERD. Twenty-nine participants were randomized to a sham procedure group and 35 to the Stretta group. The primary outcomes of interest measured were improvement in GERD symptoms and QoL. At six months of follow-up, the Stretta treatment group experienced significantly improved symptoms and QoL (61%) compared to the sham treatment group (33%). GERD QOL scores improved >50% in the treatment group subjects, compared to just 30% improvement in the sham group [43]. Interim results of a recently published randomized prospective clinical trial on a relatively young study population (mean age 34-39 years) showed statistically significant improvement in QOL and GERD symptom score post-Stretta treatment compared to PPIs at three months of follow-up. Trial results were also promising for either decrease in PPI dosage or complete cessation of PPI use after treatment [44]. Another double-blind RCT comparing the Stretta procedure vs sham group showed that tissue compliance was significantly decreased with the active Stretta procedure (17.8 ± 3.6 vs. 7.4 ± 3.4 ml/mm Hg, p < 0.05) compared to the sham procedure (14.0 ± 5.3 vs. 13.3 ± 4.30 ml/mm Hg, NS). However, no significant changes were noted in LES resting pressure or EAE at three or six months [43]. The Stretta procedure is a safe and well-tolerated treatment for GERD with <1% adverse effects noted (Table 3) [41,45].

**Table 3:** Documented potential adverse events after Stretta procedure.

Minor	Major
Chest pain, mild fever	Esophageal perforation
Small erosions	Prolonged gastroparesis
Mucosal lacerations	Mediastinal inflammation
Pneumonia	Pleural effusion
Transient nausea/vomiting	Bleeding requiring transfusion
Transient dysphagia	

*C) Concomitant Transoral Incisionless Fundoplication (c-TIF)* 

The success of the TIF technique is highly contingent on proper patient selection. Patients with a hiatal hernia of <2 cm or Hill grade I or II valves are the most likely candidates. However, because the majority of refractory GERD patients have a hiatal hernia of more than 2 cm, they do not match these strict requirements and are therefore unfit for TIF alone. The c-TIF (also known as HH-TIF: combined laparoscopic hiatal hernia repair with TIF 2.0) is a new modified approach that combines laparoscopic hiatal hernia repair and TIF in the same session. Choi et al. [48] enrolled 60 patients in their c-TIF trial, with an average age of 59.3 years and a BMI of 30.0 kg/m^2^. This study comprised people with BE, LA grade C/D esophagitis, a mean HH of 2.9 ± 1.5 cm, and HG I-IV. Some subjects had previously undergone TIF alone, Nissen fundoplication, or HH repair. Fifty-five participants in the research were using GERD medication. Upon 12 months of follow-up, statistically significant improvements in the Reflux Disease Questionnaire and GERD-HRQL (primarily heartburn and regurgitation) were reported. Only 1/7 patients (14.3%) were still on PPIs after 12 months of follow-up after c-TIF, while 2/7 patients (28.6%) were on H2 receptor blockers. A mucosal tear occurred in one patient intraoperatively. On postoperative day 1, one patient suffered a non-life-threatening upper gastrointestinal hemorrhage and ileus, and another was readmitted for gas bloat and delayed stomach emptying [48]. At six months of follow-up, another retrospective review analysis revealed statistically significant improvements in GERD-HRQL and RSI scores in 29 patients and normalization of EAE after c-TIF in 21 out of 22 patients. There were no significant adverse events associated with the operation [49].

Expanded patient eligibility can be achieved by collaboration between an interventional gastroenterologist and a surgeon. Reducing HH will restore crural sphincter defects, and TIF will help avoid adverse events associated with an LNF, such as postoperative gas/bloat and dysphagia.

Discussion

GERD impacts more than 20-40% of the US population, with at least one-third to half of those struggling from refractory GERD. This translates to more than 9 million office visits that necessitate a referral to a gastroenterologist and lost productivity and financial hardship for patients [50,51]. Endoscopic treatments such as TIF and Stretta provide a feasible, affordable, and effective alternative to PPIs for those who do not want to take them for the remainder of their lives or who do not respond to PPIs and do not want surgery. The TIF and Stretta treatments have undergone substantial improvements and evolution since their inception. The procedure provides long-term relief from GERD symptoms while also being cost-effective. As the technique has evolved, the incidence of serious adverse outcomes has also trended down. Even when TIF fails to improve these parameters, the GERD-HRQL score improves, indicating improved QoL. The underlying question is how much pH regulation is required. Is it more important than actual symptom control and QoL improvement? Patients with erosive esophagitis who are at risk of developing BE after TIF can safely resume PPIs at a lower dose and undergo endoscopic surveillance at the prescribed intervals. BE may advance to EAC at a rate of no more than 0.3% per year [52]. TIF's benefits in BE, esophageal stricture, or individuals with a previous history of esophageal, gastric, or any major abdominal surgery need to be investigated further. Concomitant Stretta, such as c-TIF, is not an option currently, and future research should look at the viability of this option. 

## Conclusions

Based on the research findings, we strongly advocate endoscopic treatments like TIF and Stretta, or modified c-TIF, as an alternative for eligible patients with refractory GERD who do not want lifelong PPIs or undergo LNF because of the risk of adverse outcomes. Endoscopic techniques have been shown to have long-term efficacy and safety. TIF and Stretta are sophisticated endoscopic procedures that successfully eliminate atypical GERD symptoms, improve GERD-HRQL score, improve esophageal healing, and reduce the need for PPIs or other antacid medications. 
